# Arthroscopic Treatment for Coronoid Process Nonunion of the Elbow

**DOI:** 10.1016/j.eats.2025.103668

**Published:** 2025-06-06

**Authors:** Olivier Bozon, Rémy Lavigne, Emeline Chapron, Cyril Lazerges, Thomas Daoulas, Bertrand Coulet

**Affiliations:** a Department of Orthopedic Surgery, Upper Limb Surgery Unit, Hospital Lapeyronie, CHU Montpellier, Montpellier, France; b Department of Orthopedic and Traumatology Surgery, Cavale Blanche Hospital, Brest, France

## Abstract

The coronoid process of the ulna (CP) plays a crucial role in elbow stability by preventing posterior dislocation and resisting posterior muscular forces. Isolated CP of the ulna nonunions are rare but can lead to chronic instability, pain, and functional impairment. Traditional management relies on open techniques with internal fixation, but these approaches are invasive and risk fragment devascularization. With advances in arthroscopy, we describe a fully arthroscopic technique for treating CP nonunions using transosseous suture fixation with no bone graft. This minimally invasive approach offers a promising alternative by preserving periarticular structures while ensuring stable fragment fixation.

The coronoid process (CP) of the ulna plays a crucial role in elbow stability by preventing posterior ulna dislocation and maintaining joint alignment. Biomechanically, it resists posterior forces of the flexor and extensor muscles. Different classifications have been proposed to describe these fractures, including the Regan and Morrey classification, which categorizes them on the basis of their height,^1^ or the more detailed O’Driscoll classification, which is determined by a computed tomography evaluation in which the fracture site, fragment size, and injury mechanism are considered.^2^ A CP fracture is a key fracture entity that can be part of complex elbow instability patterns, or, more rarely, as an isolated fracture,^3^ and can lead to various complications such as nonunions. Isolated coronoid nonunions are rare and usually asymptomatic; however, when symptomatic, they can lead to chronic instability, persistent pain, and reduced joint function, significantly impacting patients' quality of life.^4^

Elbow arthroscopy has advanced significantly in recent years, providing a minimally invasive alternative for the treatment of complex articular pathologies.^5^ Arthroscopic management of recent CP fractures has been described by several authors, either as a standalone procedure or as part of the treatment of complex trauma cases.^6^, ^7^, ^8^ This study presents a fully arthroscopic technique for managing CP fracture nonunions.

## Surgical Technique

The step-by-step description of the surgical technique is provided in Video 1.

### Patient Positioning, Arthroscopic Portals, and Exploration

The patient is positioned in the lateral decubitus position on the operating table, with the operative arm secured on an arm holder. The elbow is placed at 100° of flexion, whereas the forearm remains free, allowing full flexion-extension movements during the procedure. A nonsterile tourniquet is applied at the proximal limb and inflated to 250 mm Hg after exsanguination. Bony and soft-tissue landmarks are identified, including the olecranon, radial head, epicondyles, and the course of the ulnar nerve in the cubital groove. Twenty millimeters of normal saline solution is injected to distend the elbow capsule via the soft point, located at the center of a triangle formed by the olecranon tip, lateral epicondyle, and radial head.

**Fig 1:**
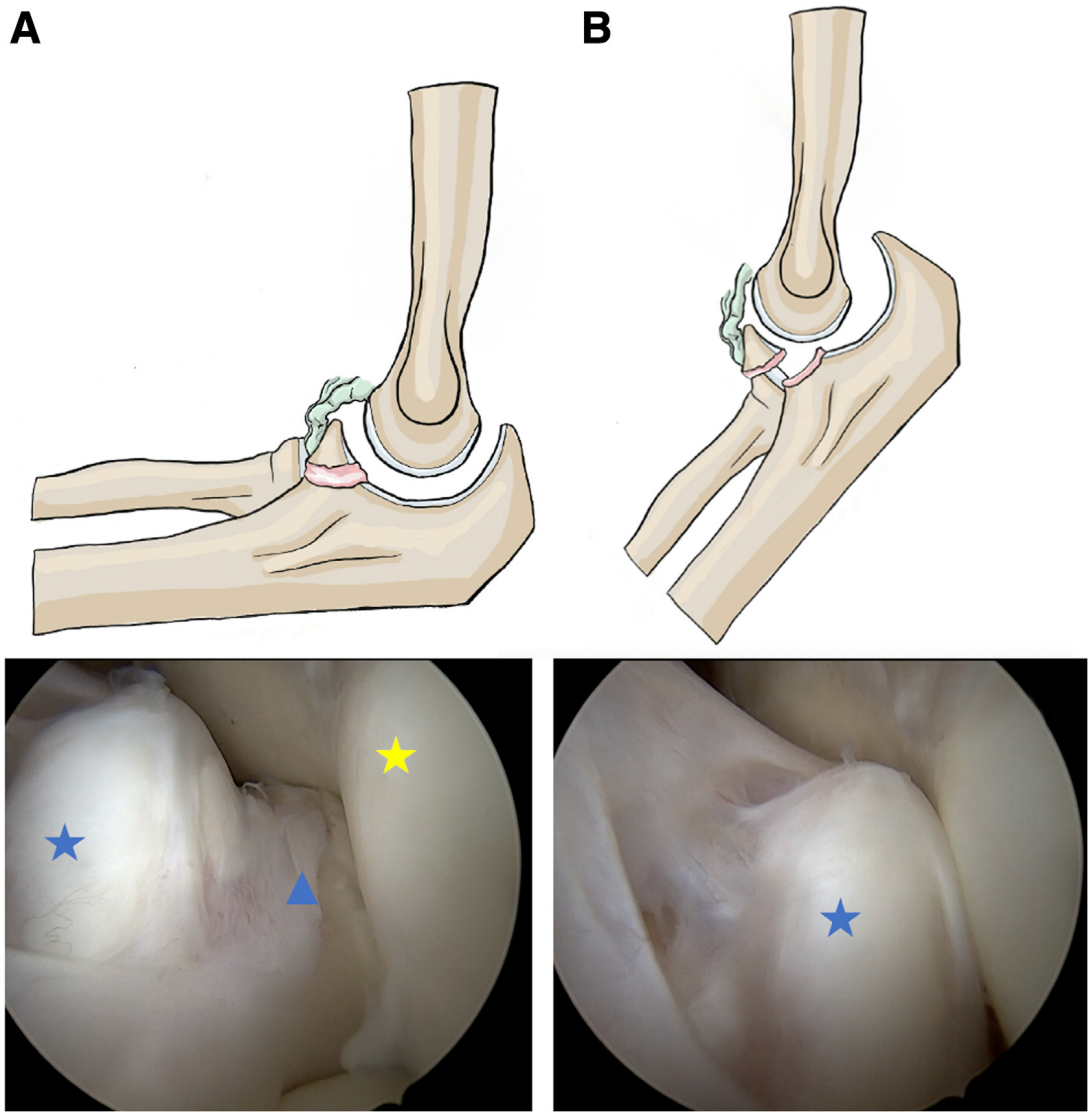
Radiographic assessment of a coronoid process nonunion and posterior displacement of the ulna in a left elbow (lateral view). (A) Persistent coronoid process nonunion is shown. (B) Posterior ulnar subluxation is shown. The patient is positioned supine with the elbow in lateral projection. Blue star: coronoid process; blue triangle: ulnar base; yellow star: humeral trochlea. This assessment confirms joint instability and the need for surgical intervention.

An anterosuperior lateral optical portal is first established using a 30° arthroscope, followed by an anterosuperior medial working portal in an in-out fashion. Joint exploration is performed. The CP is examined, and nonunion of the coronoid fragment is confirmed using a probe. The bony fragment’s size and mobility are assessed. Posterior axial instability of the ulna may be observed in extension during passive flexion-extension movements and posterior drawer testing of the ulna under the humerus (Fig 1). A stability assessment of the elbow and radial head is performed to detect any associated posterolateral rotatory instability or medial elbow instability in valgus

### Debridement and Preparation of the Nonunion Site

**Fig 2:**
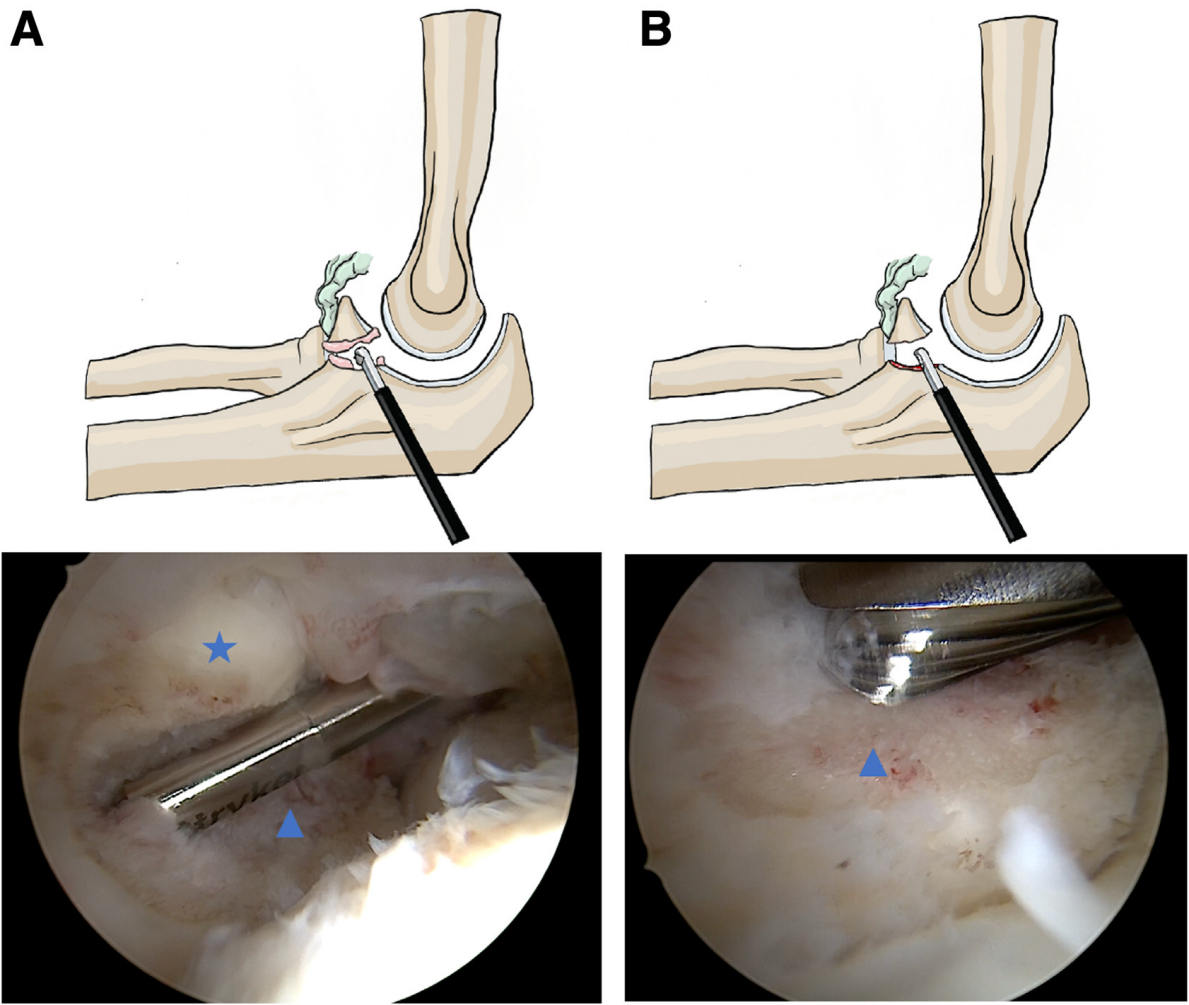
Arthroscopic debridement and burring of the nonunion site of the left elbow (patient in the lateral decubitus position, viewing through the anterolateral portal). (A) Soft-tissue debridement of the nonunion site. (B) Burring until cancellous bone is exposed. Blue star: coronoid process; blue triangle: ulnar base. This step is essential to promote biological healing and ensure fragment integration.

The nonunion site is released using an electrocoagulation probe followed by a shaver, both introduced through the medial portal. The fibrous tissue of the nonunion is debrided from both bony surfaces without causing any damage to the humeral or ulnar cartilage. Complete mobilization of the fragment, which remains attached to the anterior capsule, must be achieved and confirmed at the end of the preparation. The coronoid fossa is also cleared to prevent any secondary impingement. Bone surface preparation of the CP is performed using a 4-mm motorized burr after fibrous tissue release. Visible bleeding on both sides of the nonunion must be obtained, without causing excessive bone loss (Fig 2).

### Creation of the Ulnar Tunnel

**Fig 3:**
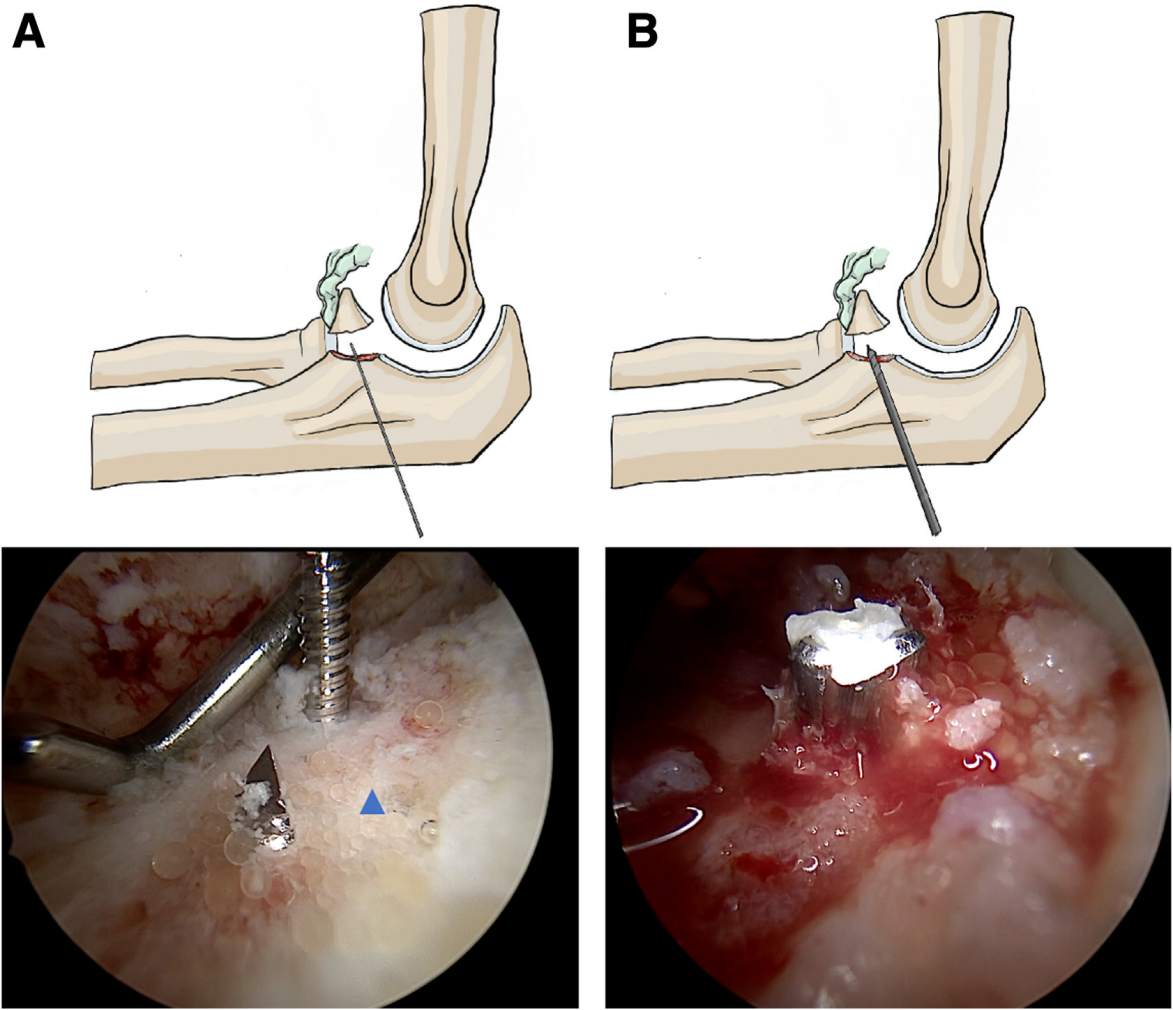
Posterior-to-anterior pinning and drilling of the ulna to prepare the tunnel for endobutton fixation (left elbow, viewing through the anterolateral portal). (A) Initial guidewire placement. (B) Drilling through the ulnar base. Blue triangle: ulnar base. Proper tunnel orientation is critical to ensure stable suture passage and avoid neurovascular injury.

A 1.4-mm diameter threaded guidewire (Asnis III; Howmedica, a Stryker Company, Kalamazoo, MI) is percutaneously inserted freehand using a power drill from the dorsal aspect of the ulnar ridge, aiming toward the center of the prepared coronoid nonunion site. A second guidewire may be inserted to help achieve optimal positioning. Once the wire is correctly placed, a 2.7-mm diameter tunnel is created using a cannulated drill over the guidewire, which is then removed. The guidewire is then removed, while the cannulated drill is left in place (Fig. 3).

### Coronoid Suture Passage

**Fig 4:**
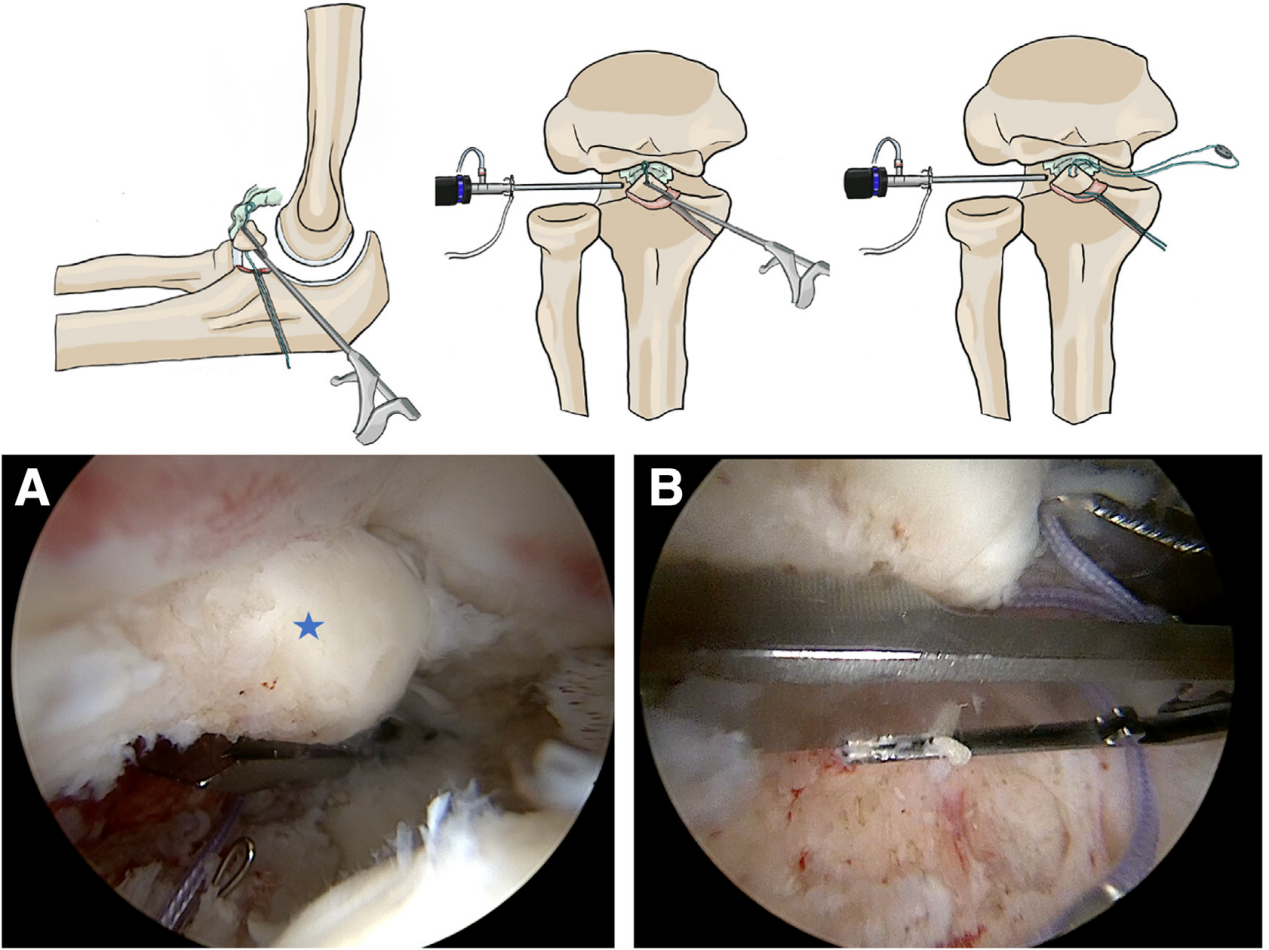
Arthroscopic suture passage through the anterior capsule and coronoid process in a right elbow (viewing from anterolateral portal). (A) Deep-to-superficial passage. (B) Superficial-to-deep technique. Blue star: coronoid process. This step allows capsular tensioning and fragment stabilization using an endobutton.

A looped shuttle wire is passed through the cannulated drill from dorsal to intra-articular and retrieved through the anteromedial working portal. A nonabsorbable No. 2 FiberWire suture (Arthrex, Naples, FL) is then shuttled using the looped wire from the anteromedial portal into the joint and through the cannulated drill, which is then removed. A Scorpion automatic suture passer (Arthrex) is used to pass the medial limb of the FiberWire suture from the deep to the superficial surface of the CP, incorporating the anterior capsule. The passed suture limb is then retrieved through the anteromedial arthroscopic portal. A titanium endobutton (Smith & Nephew, Andover, MA) is placed onto the suture. The suture limb is then passed again using the suture passer, from the superficial to the deep surface of the CP, once again incorporating the anterior capsule. The suture limb is once again retrieved through the anteromedial portal (Fig 4).

Finally, a new looped steel wire is passed through the ulnar tunnel from dorsal to intra-articular and retrieved via the anteromedial portal in order to grasp the FiberWire suture limb. The suture is then pulled through the ulnar tunnel using the looped wire and retrieved dorsally on the ulna.

### CP Fixation

**Fig 5:**
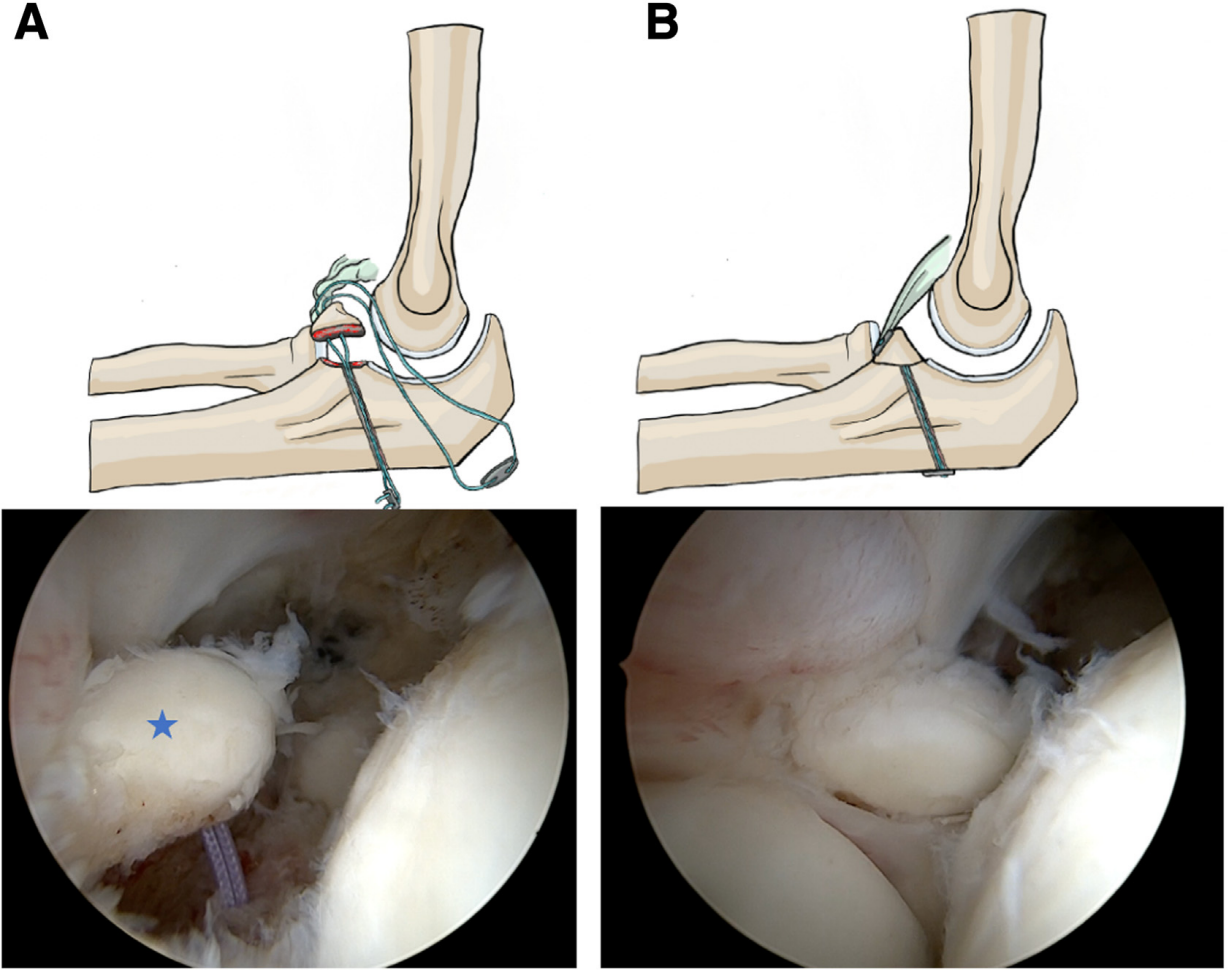
Final positioning of the endobutton before and after fixation in a right elbow (lateral decubitus position, posterior view). (A) Suture endobutton is shown before final tensioning. (B) Completed fixation. Blue star: coronoid process. Proper tensioning of the anterior capsule and stabilization of the tip are achieved without hardware prominence.

The transosseous sutures are secured to an endobutton (Smith & Nephew). The elbow is positioned at 90° of flexion, and the sutures are tied under tension over the button placed on the dorsal cortical surface of the ulna. The stability of the construct is then assessed using a probe (Fig 5).

### Postoperative Care

The patient is immobilized with a posterior brachio-antebrachio-palmar plaster splint for a duration of 6 weeks. Assisted active flexion-extension rehabilitation is initiated at 6 weeks, whereas pronosupination exercises begin immediately. Strength recovery and manual activities are resumed at 3 months.

## Discussion

Surgical management of recent CP fractures is indicated in cases of anteromedial facet fractures, associated radial head fractures (terrible triad), or fractures involving more than 50% of its height. Arthroscopic techniques may be employed in selected acute cases.^6^, ^7^, ^8^

The treatment modalities of CP nonunions are less common and remain relatively confidential, relying primarily on open approaches that combine internal fixation with bone reconstruction. Several reconstruction techniques have been described for comminuted nonunions or cases with bone loss, including reconstruction using the olecranon tip as proposed by Moritomo et al.,^9^ structural autografts,^10^ and coronoid prostheses.^11^

The arthroscopic approach to this pathology offers several advantages, as reported in Table 2. This technique has been successfully employed in the treatment of various nonunions of articular fractures, such as those of the scaphoid^12^ and capitate.^13^ In addition, it allows for both the diagnosis and management of associated lesions, including lateral collateral ligament imbrication^14^ or the removal of loose bodies. The technique is also relatively easy to perform, although precautions must be taken to avoid technical errors that could compromise the final outcome. Among the key technical pearls, performing bone debridement until visible bleeding is achieved is essential to promote healing. Intraoperative orientation must be maintained at all times, and the anterior capsule should be properly captured during fixation. When the trajectory is uncertain, the use of multiple guidewires may help secure optimal positioning. These precautions are consistent with the recommendations of Marois and Field^15^ and Hasan et al.,^16^ who emphasize the importance of thorough anatomical knowledge and meticulous arthroscopic technique to minimize the risk of complications, particularly neurologic ones. Furthermore, the absence of a need for bone grafting simplifies the surgical protocol in cases without bone loss, but adequate debridement to expose cancellous bone is crucial to facilitate healing.

However, this approach also has disadvantages and risks (Tables 1 and 2). One major challenge is the lack of a dedicated drilling guide for the ulnar tunnel, making precise drilling more difficult. This limitation can increase the risk of misplacement and potential injury to surrounding structures. In addition, the passage of sutures and ENDOBUTTON fixation require meticulous handling to prevent tangling of the sutures before passing the device, which could compromise final fixation. Failure to control this step can lead to fixation failure or elongation of the operative time.

**Table 1:** Pearls and Pitfalls

Pearls	Pitfalls
Perform bone debridement until visible bleeding is achieved	Grasping a coronoid fragment that is too small, leading to fragmentation
Use multiple guidewires if necessary to ensure proper trajectory	Tangling the sutures before passing the endobutton
Ensure proper capture of the anterior capsule	Inaccurate drilling trajectory because of lack of a dedicated guide, increasing risk of fixation failure or neurovascular injury
Maintain continuous arthroscopic orientation to avoid trajectory errors	Inadequate debridement, leaving fibrous tissue that may limit bone healing

**Table 2:** Advantages and Disadvantages

Advantages	Disadvantages
Low morbidity of the procedure	No dedicated drilling guide for the ulnar tunnel, making the technique more challenging
Possibility of diagnosing and treating associated intra-articular lesions of the elbow	Complexity of suture passage and button fixation placement
No need for bone graft in absence of bone loss	Risk of bone fragmentation when attempting to fix very small fragments
Relatively easy technique in selected cases without bone loss No need for bone graft in absence of bone loss	Subcutaneous position of the dorsal plate may cause discomfort and often requires hardware removal
Minimally invasive approach with limited soft-tissue disruption	Inherent neurologic risks of elbow arthroscopy if portals are not precisely placed

Among the pitfalls to avoid, it is crucial not to grasp a coronoid fragment that is too small, as this could lead to further fragmentation. In such cases, fixation should instead focus on soft-tissue attachment such as the anterior capsule, which can be retensioned using ENDOBUTTONs. In this particular case of tip fracture, the anterior capsule is stitched, not the bony fragment, which is only debrided. Therefore, careful preparation and mastery of the technique are essential to ensure optimal outcomes. Regarding the osteosynthesis method, the use of ENDOBUTTONs allows for capsular retensioning by passing sutures through the anterior capsule, as well as fixation of small fragments that would otherwise risk fragmentation if secured with cannulated screws. However, the dorsal positioning of the plate on the ulna can cause discomfort during weight-bearing, often necessitating subsequent removal of the subcutaneous hardware.

For postoperative management, although elbow immobilization carries a significant risk of stiffness, surgical management of CP nonunion requires immobilization in flexion-extension for 6 weeks. In the absence of associated repairs, pronosupination mobilization can begin immediately, with the elbow flexed at 90°.

## Disclosures

All authors (O.B., R.L., E.C., C.L., T.D., B.C.) declare that they have no known competing financial interests or personal relationships that could have appeared to influence the work reported in this paper.

## Supplementary Data

Video 1 Step-by-step arthroscopic surgical technique for treating coronoid process nonunion. The video demonstrates arthroscopic portal setup, debridement, tunnel drilling, suture passage, and final fixation using a transosseous suture technique. Key surgical landmarks and intraoperative findings are highlighted.
